# Thickness of subcutaneous fat is a risk factor for incisional surgical site infection in acute appendicitis surgery: a prospective study

**DOI:** 10.1186/s12893-020-01029-7

**Published:** 2021-01-04

**Authors:** Bikas Thapa, Edward Sutanto, Raju Bhandari

**Affiliations:** 1 Department of General and Gastrointestinal Surgery, Tribhuvan University Teaching Hospital, Institute of Medicine, Tribhuvan University, Maharajgung, Kathmandu, Nepal; 2 Department of Health Behavior, Roswell Park Comprehensive Cancer Center, Buffalo, NY USA

**Keywords:** Subcutaneous fat, Surgical site infection, Acute appendicitis, Ultrasonography

## Abstract

**Background:**

Incisional surgical site infection (SSI) is a significant source of postoperative morbidity resulting in increased length of stay and cost. In this study, our aim was to evaluate the association between thickness of subcutaneous fat (TSF) and incisional SSI among patients undergoing open appendectomy in low-resource settings.

**Methods:**

90 patients with acute uncomplicated appendicitis who underwent emergency open appendectomy from December 2017 to August 2018 were included in this prospective study. TSF was measured preoperatively using ultrasound. TSF and other possible predictors of incisional SSI, including body mass index and other clinical characteristics, were assessed by univariate and multivariable logistic regression analysis. Receiver operating characteristic (ROC) curve analysis evaluated the predictive value of TSF and the optimum cut-off value for TSF was determined using the Youden index.

**Results:**

The prevalence of incisional SSI was 13.3% (12/90). TSF was independently associated with incisional SSI (P < 0.001). Additionally, history of smoking (P = 0.048) was also associated with incisional SSI. A model of incisional SSI using a cut-off of 23.0 mm for TSF was moderately accurate (area under curve 0.83, confidence interval 0.70–0.97; sensitivity 83.3%; specificity 76.9%).

**Conclusions:**

The study demonstrated that TSF, as evaluated by ultrasound, is a predictor in the development of incisional SSI in patients with acute appendicitis undergoing open appendectomy. These findings suggest that ultrasound is useful both for the evaluation of TSF and the prediction of incisional SSI risk factor in low-resource settings.

## Introduction

Surgical site infection (SSI), a leading healthcare-associated infection reported in low- and middle-income countries, remain a significant clinical challenge as it is associated with substantial mortality and morbidity [1, 2]. SSI prolonged hospitalization, diminished quality of life, and imposed substantial cost burden [3, 4]. SSI, including incisional SSI and organ/space SSI, is the most frequent postoperative complication of acute appendicitis, which is a common surgical emergency worldwide [5]. Several systematic reviews have noted that there is a higher burden of SSI in low-income countries [1, 6]. SSI is especially common after open appendectomy, which remain the most common surgical approach for acute appendicitis in low-income countries [7]. Thus, identification of risk factors for SSI is important, especially in the context of low-resource settings, as it could contribute towards the development of interventions that may reduce SSI incidence among patients undergoing open appendectomy.

Obesity is an established risk factor for SSI [8–10]. Several mechanisms have been hypothesized by which obesity increases the incidence of SSI, including impaired immune function, diminished oxygen tension within surgical wounds, and poor tissue penetration for perioperative antibiotics [11]. Although body mass index (BMI) is typically used to measure obesity, there is a limitation on how precisely it describes body composition [12, 13]. While BMI may be appropriate for population-wide studies, its utility in clinical setting to assess perioperative risk may be limited as it does not accurately measure adiposity [13]. Targeted measure of body composition, such as thickness of subcutaneous fat (TSF) at surgical site, may improve assessments for SSI risk instead.

Several studies have reported TSF as an independent risk factor of incisional SSI in colorectal surgery [14, 15]. In these retrospective studies, preoperative TSF was evaluated using computed tomography (CT) scan. However, CT scan for patient presenting with acute appendicitis may not be feasible in low-resource settings due to the high cost and low availability in these settings. Ultrasound, a simple and non-invasive alternative modality, has been demonstrated to be able to measure TSF reliably [16, 17]. Thus, we aimed to evaluate the association between thickness of subcutaneous fat, through preoperative ultrasound measurements, and incisional SSI among patients undergoing open appendectomy in low-resource setting.

## Methods

### Patients

In this prospective observational study, adult patients (age ≥ 16 years) who underwent emergency open appendectomy with American Society of Anesthesiology score of < 4 at Tribhuvan University Teaching Hospital, Kathmandu, Nepal from December 2017 to August 2018 were identified for inclusion in this study. Patients with complicated appendicitis (gangrenous or perforated appendicitis), appendicitis associated with other abdominal pathologies (ascites, peritonitis, bowel obstruction, and abdominal and pelvic malignancies), diabetes mellitus, currently taking immunosuppressants, and underwent emergency open appendectomy by midline incision were excluded from the study.

All patients presenting to emergency department with clinical diagnosis of acute appendicitis underwent ultrasound of abdomen and pelvis to measure TSF at McBurney’s point. Prior to open appendectomy, TSF was measured in between skin and external oblique aponeurosis using a Toshiba ultrasound with a 7.5 MHz high-frequency linear probe. All patient underwent open appendectomy by incision (either gridiron or lanz incision) at McBurney’s point. In the case of intraoperative finding of complicated appendicitis, either perforated or gangrenous with or without fecal contamination, thorough wash of abdominal cavity was done using normal saline and the use of intra-abdominal drain was left in accordance with surgeon’s preference. As mentioned, complicated cases, which may require drain, were excluded from the study. Neither subcutaneous drain nor subcutaneous flap were used in any of the cases. All patients were given intravenous prophylactic antibiotics using third-generation cephalosporin, with administration being started 30 min before surgery, and continued until 24 h after surgery. Intravenous antibiotics were then switched into oral antibiotics for the remainder of treatment. Wound inspection to evaluate incisional SSI was done on second, seventh, fifteenth, and thirtieth postoperative day.

Diagnoses of incisional SSI were made in accordance with the guidelines of the Centers for Disease Control and Prevention, Public Health Service, US Department of Health and Human Services [18]. Incisional SSI can be further subclassified into superficial and deep incisional SSI. Superficial incisional SSI was defined as occurring within 30 days of surgery, infection involving only skin or subcutaneous tissue of an incision, and at least one of the following: (1) purulent drainage; (2) organisms isolated from an aseptically obtained culture of fluid or tissue from the superficial part of an incision; or (3) signs or symptoms of infection, including pain, tenderness, localized swelling, redness, heat, and the superficial part of an incision deliberately opened by the surgeon, unless the incision was culture-negative. Deep incisional SSI was defined as infection involving the fascia of the incision and at least one of the following: (1) purulent drainage deep in an incision; (2) spontaneous dehiscence or deliberate opening of a deep incision by a surgeon in a patient with the following signs or symptoms: fever (> 38 °C) and localized pain or tenderness, unless the site is culture-negative; or (3) an abscess or other evidence of infection involving a deep incision is found on direct examination, during reoperation, or by histopathologic or radiologic examination. Diagnosis of an incisional SSI were made by a surgeon or attending physician.

Other variables of interest recorded for each patients were as followed: age, sex, weight, height, smoking habits, alcohol use, and duration of surgery. Postoperative complications among these patients were graded according to the Clavien-Dindo classification [19]. The sample size was calculated on the basis of a previous study which recorded prevalence of SSI following open appendectomy as 6.25% [20]. With 95% confidence interval (CI) and permissible error of 5%, the minimum sample size was calculated as n = 90.

### Statistical analysis

Continuous data were tested for normality using the Shapiro–Wilk test. Normally distributed data were analyzed using unpaired t-test and presented as mean ± standard deviation. Non-normally distributed data were analyzed using the Mann–Whitney U test and presented as median and interquartile range (IQR). Categorical data were analyzed with Fischer’s exact test and presented as frequencies and percentages. Univariate and multivariable logistic regression analyses were performed to identify predictors of incisional SSI. Only variables with P < 0.1 in univariate analysis were assessed by multivariable logistic regression analysis. Multicollinearity was assessed using a variance inflation factor (VIF) with a cut off value of 5 [21]. The Hosmer–Lemeshow test was reported for the model.

Predictive accuracy was assessed by receiver operating characteristic (ROC) curve analysis. The receiver operating characteristic (ROC) curve is the plot of sensitivity versus 1-specificity while the area under the curve (AUC) is an effective and combined measure of sensitivity and specificity that describes accuracy or the inherent validity of diagnostic tests [22]. To determine the optimal cut-off values for TSF, the Youden index (sensitivity + specificity − 1) was calculated, and the values for the maximum of the Youden index was considered as the optimal cut-off points [22]. All statistical analyses were performed in Stata SE version 14.2 (StataCorp, College Station, TX, USA). All tests were two-tailed and considered significant at P < 0.05.

## Results

The study cohort comprised 90 patients (57 men, 63.3%) with the mean age of 31.7 years and age distribution from 16 to 65 years. The mean BMI was 22.5 kg/m^2^ (ranging from 16.6 kg/m^2^ to 29.5 kg/m^2^) while the mean TSF was 18.7 mm (ranging from 4.0 to 50.0 mm). The prevalence of smoking and alcohol use in our study cohort was the same, which is 6.7% (6/90). 9 (10.0%) patients had surgery with duration more than 60 min. The prevalence of incisional SSI in this cohort was 13.3% (12/90). None of the patients had deep incisional or organ space SSI. All complications were classified as Clavien-Dindo Grade 1.

Table 1 compared characteristics between patients who developed SSI and those who did not. Median TSF was 30.0 mm in patients who developed incisional SSI and 15.0 mm in those who did not (P < 0.001). While there was no difference in age, sex, and alcohol use between patients who developed incisional SSI and those who did not, there was a significant difference in BMI, smoking, and duration of surgery. The mean BMI in patients who developed incisional SSI was 23.8 kg/m^2^, compared to mean BMI of 22.2 kg/m^2^ among patients who did not develop incisional SSI. One-fourth (25.0%) of patients who developed incisional SSI were smokers, yet only 3.8% of patients who did not develop incisional SSI were smokers. Half (50.0%) of the patients who developed incisional SSI had ≥ 60 min surgery duration, compared to 3.8% of patients who did not develop incisional SSI had ≥ 60 min surgery duration.

**Table 1:** Characteristics of the study groups

Variable	Surgical site infection		*P* value
	Present (n = 12)	Absent (n = 78)	
Age (years)	30 (22–36)	28 (21–38)	0.967
Male sex	5 (41.7)	52 (66.7)	0.115
Body mass index (kg/m^2^)	23.8 ± 3.1	22.2 ± 2.5	0.049
Thickness of subcutaneous fat (mm)	30 (23–43)	15 (10–20)	< 0.001
Smoking	3 (25.0)	3 (3.8)	0.029
Alcohol use	2 (16.7)	4 (5.1)	0.181
Duration of surgery ≥ 60 min	6 (50.0)	3 (3.8)	< 0.001

Values are reported as n (%), mean ± standard deviation, or median (interquartile range)

To investigate associations between individual risk factors and prevalence of incisional SSI, factors (TSF, BMI, smoking, and duration of surgery) found to be P < 0.1 in univariate analysis were subjected to multivariable analysis. VIF for BMI and duration of surgery variables were 15.06 and 15.40 respectively, indicating that multicollinearity was an issue. These two variables were subsequently dropped from the final model. In addition to the univariate analysis, Table 2 reported the final multivariable analysis which showed that TSF was independently associated with incisional SSI (P < 0.001). Additionally, smoking was also independently associated with incisional SSI (P = 0.048). VIF were less than 2 for all variables in the final model.

**Table 2:** Risk factors for incisional surgical site infection in univariate and multivariable analysis

Variable	Univariate analysis		Multivariable analysis	
	OR (95% CI)	*P* value	OR (95% CI)	*P* value
Age	0.99 (0.94–1.05)	0.789	–	–
Male sex	2.80 (0.81–9.68)	0.104	–	–
Body mass index	1.25 (0.99–1.58)	0.055	–	–
Thickness of subcutaneous fat	3.46 (1.80–6.68)	< 0.001	3.52 (1.75–7.08)	< 0.001
Smoking	8.33 (1.46–47.63)	0.017	13.65 (1.02–182.97)	0.048
Alcohol use	0.27 (0.04–1.67)	0.159	–	–
Duration of surgery ≥ 60 min	25.00 (4.97–125.81)	< 0.001	–	–

Hosmer–Lemeshow goodness of fit (P = 0.350) for multivariable analysis

**Fig. 1:**
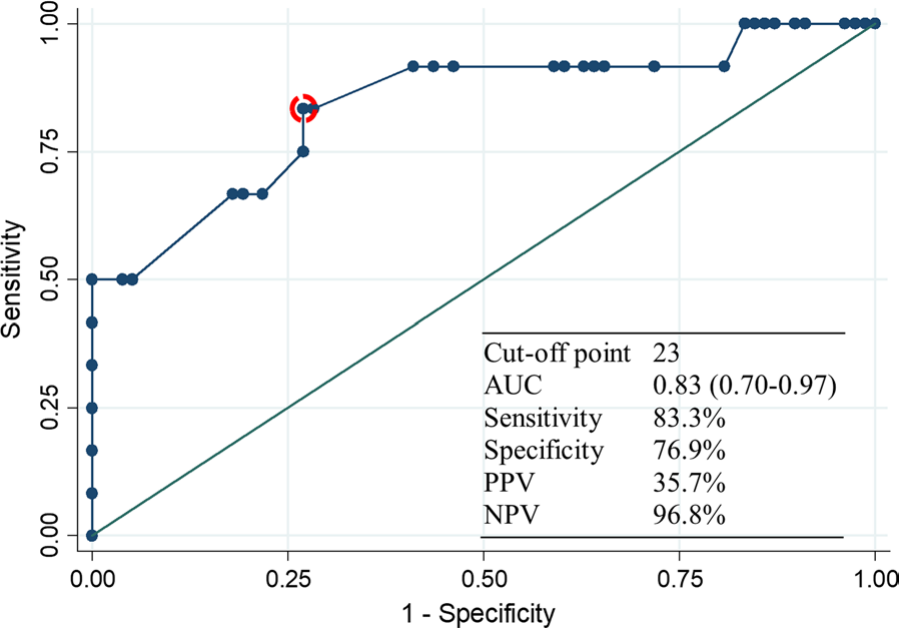
The ROC curve shows predictive value of thickness of subcutaneous fat for incisional surgical site infection. *AUC* area under the curve, *NPV* negative predictive value, *PPV* positive predictive value, *ROC* receiver operating characteristic

ROC curve was constructed to determine predictive value of TSF for incisional SSI in patients undergoing open appendectomy (Fig. 1). The area under the curve (AUC) was 0.83 (95% CI 0.70–0.97). Using Youden index, the optimal TSF cut-off value for maximum sensitivity and specificity was 23 mm (sensitivity, 83.3%; specificity, 76.9%; positive predictive value, 35.7%; negative predictive value, 96.8%).

## Discussions

In this prospective study, our primary aim was to evaluate the association between TSF and incisional SSI in patients undergoing open appendectomy. First, the prevalence of incisional SSI in our patient population was 13.3%, which is slightly over half of the prevalence of SSI following abdominal surgery reported in our center in 2011 [23]. A recent meta-analysis assessed that, while the global overall incidence of SSI after appendectomy was 7.0%, the incidence of SSI was higher in low-income countries (11.1%; compared to high-income countries: 6.2%) and open appendectomy (11.0%; compared to laparoscopic appendectomy: 4.6%) [6]. Further, a systematic review that focused on low and middle Human Development-Index Countries reported the overall SSI rate was 17.9% in open appendectomy [7], thus the rate of SSI in our patient population was consistent with these two studies.

Second, we found that TSF is a predictor for incisional SSI in patients undergoing open appendectomy. This finding confirms what other studies have shown that TSF was independently associated with SSI in a variety of surgical procedures, including Crohn’s disease surgery, elective colorectal surgery, abdominal hysterectomy, and cervical spine fusion surgery [14, 15, 24, 25]. In our study, BMI was shown to be linearly related with TSF as evidenced by multicollinearity. This is consistent with previous study that reported BMI was correlated with TSF [15, 24]. However, BMI has increasingly been recognized as a rather poor indicator of percent of body fat and does not capture on distribution of fat in the body [26]. Indeed, several studies did not find association between BMI and SSI [14, 15, 24, 27], thus showing a mixed result whether BMI measurement can provide utility in predicting wound infection risk after surgery. Our study indicates that TSF can be a more useful indicator in predicting incisional SSI rather than BMI.

Third, the optimal cut-off value for TSF in our patient population was 23 mm. Similarly, two studies reported cut-off value for TSF of 20 mm and 30 mm for patients who developed incisional SSI after undergoing elective colorectal surgery and abdominal hysterectomy, respectively [15, 25]. However, other study has reported a markedly lower cut-off TSF value of 10.2 mm for patients undergoing Crohn’s disease surgery [14]. This difference can be explained as Crohn’s disease is a type of wasting disease and thus differs from other diseases [14]. This highlights the need for a differential cut-off value for TSF depending on diseases involved. Future study should seek to confirm appropriate cut-off value for TSF for other diseases.

Similar with BMI, duration of surgery was linearly related with TSF in our study. Due to increased TSF at the surgical site, surgeon may need longer operative time to create longer incision, wider dissection, and increased retraction owing to increased difficulty in the surgery [24]. Additionally, we found smoking was independently associated with incisional SSI. The link between smoking and risk of SSI has been substantiated by multiple systematic reviews [28–30]. Several pathophysiological mechanisms have been hypothesized to explain this phenomenon, which are attenuation of inflammatory healing response and reduced reparative cell functions [31, 32].

Unlike previous studies that used CT scan [14, 15, 24], we used ultrasound to measure TSF in our patient population. While studies have noted CT scan have higher sensitivity and specificity in the diagnosis of appendicitis, ultrasound may offer a role as an alternative modality due to its higher availability, no ionizing radiation exposure, and lower cost [33, 34]. In our center, ultrasound was routinely performed for patients with clinical suspicion of acute appendicitis. As previous study has suggested that obtaining a CT scan just to measure TSF is not recommended [15], our study supports the use of ultrasound as an alternative modality to measure TSF for incisional SSI risk prediction in patients undergoing open appendectomy.

While the prospective nature of this study is a strength, there are several study limitations that could provide an opportunity for future exploration. First, ultrasound is operator-dependent thus a standardization for the measurement technique is necessary. In our study, although different radiology residents performed the ultrasound, the TSF measurement was a significant predictors for incisional SSI. Second, there are several other risk factors of SSI that were not evaluated in this study, including anemia, preoperative level of C-reactive protein, patient frailty, and procalcitonin [14, 21, 28, 35]. Third, we did not collect data on other probable surgical complications, including remote site infections, and excluded complicated appendicitis cases from our study. Future studies should explore the association of TSF with other surgical complications and include complicated appendicitis with larger sample size. Lastly, because the patient population in this study was Asian and a previous study has noted that the Asian population have distinct characteristics for obesity [36], future research is needed to confirm our findings in different populations.

## Conclusion

This study demonstrated that TSF, as evaluated by ultrasound, is a predictor in the development of incisional SSI in patients with acute appendicitis undergoing open appendectomy. Our results suggest that ultrasound is useful both for the evaluation of TSF and the prediction of incisional SSI risk in low-resource settings. Therefore, we recommend surgical technique that minimize the risk of incisional SSI, including delayed primary closure, minimal handling of soft tissues, minimal use of cautery at subcutaneous plane, and the use of subcutaneous drain, for patients with TSF > 23 mm undergoing open appendectomy.

## Data Availability

The dataset used for the current study is available from the corresponding author on reasonable request.

## Abbreviations

AUC: Area under the curve
BMI: Body mass index
CI: Confidence interval
CT: Computed tomography
IQR: Interquartile range
NPV: Negative predictive value
OR: Odds ratio
PPV: Positive predictive value
ROC: Receiver operating characteristic
SSI: Surgical site infection
TSF: Thickness of subcutaneous fat
VIF: Variance inflation factor
